# Protein crystallography and drug discovery: recollections of knowledge exchange between academia and industry

**DOI:** 10.1107/S2052252517009241

**Published:** 2017-06-29

**Authors:** Tom L. Blundell

**Affiliations:** a Department of Biochemistry, University of Cambridge, 80 Tennis Court Road, Cambridge CB2 1GA, England

**Keywords:** protein structure, protein crystallography, fragment-based structure-guided drug discovery, disease, cancer

## Abstract

The development of structure-guided drug discovery is a story of knowledge exchange where new ideas originate from all parts of the research ecosystem. Dorothy Crowfoot Hodgkin obtained insulin from Boots Pure Drug Company in the 1930s and insulin crystallization was optimized in the company Novo in the 1950s, allowing the structure to be determined at Oxford University. The structure of renin was developed in academia, on this occasion in London, in response to a need to develop antihypertensives in pharma. The idea of a dimeric aspartic protease came from an international academic team and was discovered in HIV; it eventually led to new HIV antivirals being developed in industry. Structure-guided fragment-based discovery was developed in large pharma and biotechs, but has been exploited in academia for the development of new inhibitors targeting protein–protein interactions and also antimicrobials to combat mycobacterial infections such as tuberculosis. These observations provide a strong argument against the so-called ‘linear model’, where ideas flow only in one direction from academic institutions to industry. Structure-guided drug discovery is a story of applications of protein crystallography and knowledge exhange between academia and industry that has led to new drug approvals for cancer and other common medical conditions by the Food and Drug Administration in the USA, as well as hope for the treatment of rare genetic diseases and infectious diseases that are a particular challenge in the developing world.

## Abstract 2

Structure-guided drug discovery is a story of applications of protein crystallography and knowledge exchange between academia and industry. It has led to new drug approvals by the Food and Drug Administration in the USA and hope for treatment of rare genetic diseases and infectious diseases in the developing world.

## 1. Introduction   

In this review, on the occasion of the award of the 2017 Ewald Prize by the IUCr, I present a personal view of the relationship between academia and industry in the development of structure-guided drug discovery. It is a story of using protein crystallography to make new medicines, but also one of knowledge exchange in emerging research ecosystems. It emphasizes the importance of original ideas from all parts of the ecosystem and provides a strong argument against the so-called ‘linear model’, where ideas flow only in one direction from academic institutions to industry. It involves not only those who are academics and entrepreneurs but also those who see the importance of science in society. It begins with ­a discussion of the contributions of J. D. Bernal, Dorothy Crowfoot Hodgkin and Max Perutz, who worked on crystalline proteins in the 1930s but who recognized the importance to medicine and biology of understanding protein structure, as well as the potential social and economic impact of science. These three amazing people influenced us all not only in our science but in our understanding of its social function. The story records the development of structure-guided drug discovery from the personal view of the author with a focus on new chemical entities rather than biologics, but reflects parallel developments in thinking about the design of new medicines that occurred in academia and in industry in many places throughout the developing and developed world.

## 2. J. D. Bernal, Dorothy Crowfoot Hodgkin and protein cystallography   

Protein crystallography began in Cambridge in the early 1930s when Bernal, known as Sage, received crystals of pepsin, brought to him by Dr G. Millikan and prepared by Dr Philpot in the laboratory of Theodor Svedberg in Uppsala. Bernal’s laboratory was multidisciplinary, working not only on the diffraction of crystals, deriving from his time with Bragg at the Royal Institution, but also on the structure of materials and water. The crystals of pepsin were in the form of ‘perfect hexagonal bipyramids up to 2 mm in length, of axial ratio *c*/*a* = 2.3 ± 0.1’. Bernal & Crowfoot (1934 ▸) examined them in their mother liquor, where they appeared ‘moderately birefringent and positively uniaxial, showing a good interference figure’. The birefringence rapidly diminished on exposure to air and the X-ray diffraction pattern of the pepsin crystals was very poor. Bernal and Crowfoot realised that it was likely that the crystals needed to be hydrated to retain order, and tried keeping the crystals of pepsin sealed in a capillary with the mother liquor used in the crystallization experiment; they obtained a beautiful diffraction pattern, indicating that water was required for the retention of protein architecture. Bernal and Crowfoot were remarkably accurate when they wrote in 1934 in *Nature*

At this stage such ideas are merely speculative, but now that a crystalline protein has been made to give X-ray photographs, it is clear that we have the means of checking them by examining the structure of all crystalline proteins, arriving at far more detailed conclusions about protein structure than previous physical or chemical methods have been able to give

(Bernal & Crowfoot, 1934 ▸). However, it took nearly 20 years to make this prediction become reality!

### 2.1. Dorothy Crowfoot Hodgkin and insulin in the 1930s   

After working with Bernal in Cambridge, Dorothy Crowfoot (later Hodgkin) returned to Oxford, enthused by the idea of protein crystallography and encouraged by Bernal to start her work on insulin. Diabetes symptoms had been observed for more than three hundred years, and had been known as the ‘pissing evile’ for even longer. The disease was understood to be aggravated by sugar, and in 1869 Paul Langerhans discovered that insulin was produced in the beta cells of the pancreas. After intense efforts in the following years, Frederick Banting, a physician, and his student Charles Best in Toronto, Canada discovered the hormone insulin in the pancreatic extracts of dogs, and showed that injection of the hormone lowered blood glucose levels. Banting and Best’s work was further developed for the treatment of human diabetes by the Canadian chemist James B. Collip and the Scottish physiologist J. J. R. Macleod.

**Figure 1:**
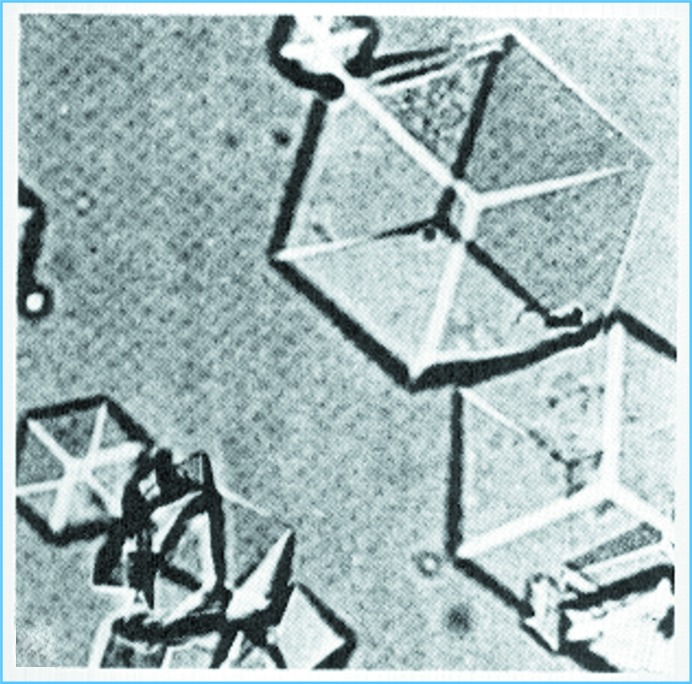
Rhombohedral insulin crystals used in the treatment of diabetes and in the determination of the structure of insulin.

The insulin used for the treatment of diabetics was purified by crystallization, a process first established in 1926. After efforts to obtain X-ray diffraction of the crystals using the powder diffraction method failed, success with the pepsin single-crystal X-ray diffraction pattern suggested to Dorothy that insulin crystals that diffract to high resolution might be produced if kept wet. The work of David Aylmer Scott at the Connaught Laboratories in Toronto, where Banting was based, also demonstrated the importance of zinc salts. Dorothy used a variation of the Scott crystallization procedure at pH 6.2–6.5 (Crowfoot, 1935 ▸), where the solution was cooled very slowly from 50° to room temperature over a period of days to produce large crystals, a method that was still used in the 1960s when I joined the Laboratory of Chemical Crystallography in Oxford where the Hodgkin group was based. The crystals formed rhombohedra, as shown in Fig. 1 ▸. Thus, from the very beginning of insulin protein crystallography, knowledge about crystallization was freely exchanged between the pharmaceutical industry and academia. Dorothy Hodgkin obtained her insulin from Messrs Boots Pure Drug Co. Ltd!

### 2.2. Max Perutz, Cambridge and early work on haemoglobin   

In 1936, Max Perutz left Vienna to seek Sage in Cambridge. Perutz records that he asked him: ‘How can I solve the secret of life?’. He replied: ‘The secret of life lies in the structure of proteins, and there is only one way of solving it and that is by X-ray crystallography’ (Perutz, 1997 ▸). However, in 1937, a year into his doctoral research, Max Perutz was bored with the use of X-ray crystallography to study mining waste, solving the crystal structures of mineral fragments in slag heaps. A biochemist cousin asked Perutz: ‘Why don’t [you] take on haemoglobin?’. The physiologist Gilbert Adair gave him some crystals of horse haemoglobin, from which he obtained good-quality X-ray diffraction patterns. In 1938, Felix Haurowitz demonstrated that on exposure to air the beautiful purple hexagonal crystals of horse deoxyhaemoglobin dissolved to give new monoclinic needle crystals, which had the scarlet colour of arterial blood (Haurowitz, 1938 ▸). Max published the first X-ray diffraction pictures of haemoglobin in *Nature* with Bernal and Fankuchen (Bernal *et al.*, 1938 ▸), showing some diffraction at the very high resolution of 2 Å. As Max Perutz later noted, ‘This was the prototype of allosteric enzymes exhibiting cooperative substrate binding and feedback inhibition for metabolic control’. Perutz’s research programme to solve the structure of haemoglobin would take 22 years.

### 2.3. Bernal and the function of science in society   

Both Dorothy Hodgkin and Max Perutz were influenced in their science by J. D. Bernal in the 1930s. However, I have to admit to having read Bernal’s *History of Science* (Bernal, 1954 ▸) and his *Social Function of Science* (Bernal, 1938 ▸) before I knew about their work in protein crystallography. Bernal had broad-ranging interests, not only in the crystallography of biological molecules but also of inorganic materials and the structure of water, which probably made him sensitive to the likely dependence of the pepsin crystal structure on the water-rich mother liquor. He also had an active involvement in social issues and politics; these came together in the *Social Function of Science*, where he asked ‘what is the function of science in society?’. His view was that it was an ‘integral part both of material and economic life and of ideas which guide and inspire it’ and ‘the means of satisfying our material needs and the ideas which enable us to understand, to co-ordinate, and to satisfy our needs in the social sphere’.

Bernal strongly championed the idea that many advances in basic science could equally well be made on subjects of industrial interest. Thus, his role as a scientific advisor in the Second World War influenced him to extend his interest in water and organic molecules to begin a study of cement. He later saw the interactions of water with silicates in very much the same way as he saw the interactions of water with proteins; he researched both in the same laboratory in the Department of Physics at Birkbeck College, London. I was appointed as his successor to the Bernal Chair at Birkbeck in 1976, nearly 40 years after his appointment, and found a very active research group involved closely with Blue Circle and other cement manufacturers, looking at the detailed hydration process. I also found an active research involvement in proteins, many of which have proved to be of medical interest. Interactions between science, society and industry were thus established at a very early stage in the development of crystallography.

## 3. Exchanging ideas on protein structure between academia and industry in the 1950s and 1960s   

**Figure 2:**
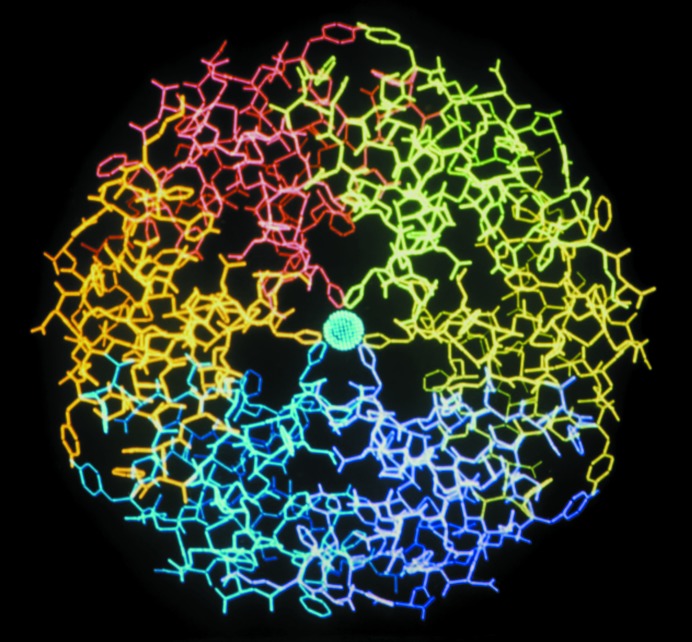
The insulin hexamer defined by X-ray analysis by Adams *et al.* (1969 ▸). The hexamer has 32 symmetry and is viewed along the threefold axis, on which two zincs are found. 2-Zinc-insulin hexamers are found in insulin-storage granules in beta cells of the islets of Langerhans and are used in crystalline forms of insulin used to treat diabetes.

Dorothy Hodgkin had the confidence to continue working on insulin for 35 years before the three-dimensional structure was solved (Fig. 2 ▸; Adams *et al.*, 1969 ▸; Blundell *et al.*, 1971 ▸). I joined the Department of Chemical Crystallography, where Dorothy was based, for an undergraduate project in 1963, and then a Part II and PhD, joining the insulin group comprising Guy and Eleanor Dodson and Margaret Adams in 1967. I found in the insulin research a remarkable interdependence between the academic study and the industrial applications. We all visited Novo, the Wellcome Foundation and Eli Lilly regularly for discussions on the production and crystallization of insulin. Jorgen Schlichtkrull of Novo had developed the theory and practice of the crystallization of insulin (Schlichtkrull, 1958 ▸) in order to obtain crystals of uniform size and of the form with two zincs per hexamer, known as 2-zinc-insulin. These had predictable half-lives in circulation when used to treat diabetics, before dissociating to insulin monomers that bound the insulin receptors and mediated the uptake of glucose. From the material supplied by Novo and the other companies and the methods developed for crystallization by Schlicht­krull, we were able to obtain beautiful diffraction patterns and solve the structure. Schlichtkrull also produced 4-zinc-insulin, which led to long-lasting insulins. Thus, my first experience of protein crystallography was an example of knowledge exchange between industry and academia, where much of the innovative work was performed in industry. The Oxford insulin group, led by Guy and Eleanor Dodson, with M. Vijayan, Margaret Adams, Ted Baker and myself, defined the molecular structure of insulin (Adams *et al.*, 1969 ▸; Blundell *et al.*, 1971 ▸), which led to hypotheses on the receptor binding and storage of insulin (Blundell *et al.*, 1972 ▸; Pullen *et al.*, 1976 ▸). This structure also illuminated the work carried out in Novo on the engineering of better insulins, and further active discussions and collaborations evolved with the companies. Some of this was with the three groups involved in the synthesis of insulin in New York (Katsoyannis), Aachen (Helmut Zahn) and Shanghai, but much of this is now achieved using recombinant insulins for the treatment of diabetes. The work continued after the mid-1970s in Guy Dodson’s laboratory in York, and has involved collaboration with Novo as well as with an international consortium from the USA and Australia on receptor structure and interactions (Menting *et al.*, 2013 ▸). This research has been carried further by Marek Brzozowski after Guy Dodson’s death in 2012.

In Cambridge, Perutz pioneered new methodologies in protein crystallography in his work on haemoglobin, including the method of isomorphous replacement for the solution of the phase problem (Green *et al.*, 1954 ▸). John Kendrew began to work on the protein structure of myoglobin later than Perutz, but solved the structure in 1957 (Kendrew *et al.*, 1960 ▸; Kendrew, 1996 ▸) using the methodologies that he and Perutz had developed. When Perutz finally caught his first glimpse of haemoglobin at 5.5 Å resolution (Perutz *et al.*, 1960 ▸), he was awestruck. ‘I finally saw this thing I had been working on for 22 years. It was like reaching the top of a mountain after a very hard climb and falling in love at the same time’, Perutz recalled. ‘That intensity of joy – maybe you find it only in science, when nature reveals its great secrets’.

The crystal structures of many variants and chemical states allowed Perutz to understand the allosteric mechanisms of haemoglobin activity, known as positive cooperativity, whereby binding of a molecule of oxygen to one protomer of the α_2_β_2_ heterotetramer allows oxygen molecules to be recruited by the other protomers with increased affinity. The structure of haemoglobin explained the molecular basis of sickle-cell anaemia, a disease resulting from a single-residue mutation, in which Perutz had a long-standing interest (Perutz *et al.*, 1951 ▸, 1978 ▸).

The structure of human haemoglobin and the binding of the deoxygenated form of the protein to 2,3-diphosphoglycerate (DPG) also led to interest by companies, including the Wellcome Foundation, in designing compounds which should bind to the deoxy conformation and stabilize it for clinical use. The compounds were designed to bind to the DPG site and thereby promote oxygen liberation (Beddell *et al.*, 1976 ▸). This was one of the first attempts to use structure to design novel molecules. Later, Beddell *et al.* (1984 ▸) went on to design substituted benzaldehydes that bind preferentially to the oxy conformation of human haemoglobin at a site between the amino-terminal residues of the α sub­units, thus stabilizing the oxygenated form of haemoglobin and thereby increasing its oxygen affinity. These molecules were designed to inhibit the sickling of sickle erythrocytes.

## 4. Enzyme crystallography, comparative modelling and drug discovery   

### 4.1. From pepsins to renin and the design of anihypertensives   

Although the structure of pepsin was solved by Andreeva *et al.* (1984 ▸), the crystal structure of porcine pepsin, began by Bernal and Crowfoot Hodgkin in 1932, was eventually solved in 1990 (Cooper *et al.*, 1990 ▸); it had proved to be an easily crystallizable form but one that was not easily susceptible to X-ray analysis owing to its ∼300 Å hexagonal axis. In the meantime, the pepsins had become of commercial interest because of their relationship to chymosin or renin, used in making cheese. Indeed there was an active interest in industry, for example Pfizer, in extracting and characterizing similar enzymes from fungi for cheesemaking. The first structures of the pepsin superfamily came from these initiatives, and much of the biochemistry and the understanding of the structure and function of, for example, endothiapepsin, the enzyme from *Endothia parasitica*, derived from this interest (for a review, see Whitaker, 1970 ▸). The structures of the enzymes endothiapepsin (Subramanian *et al.*, 1977 ▸), rhizopuspepsin (Subramanian *et al.*, 1977 ▸) and penicillopepsin (James *et al.*, 1977 ▸) proved to be a useful basis for understanding the mechanism of aspartic proteinases.

**Figure 3:**
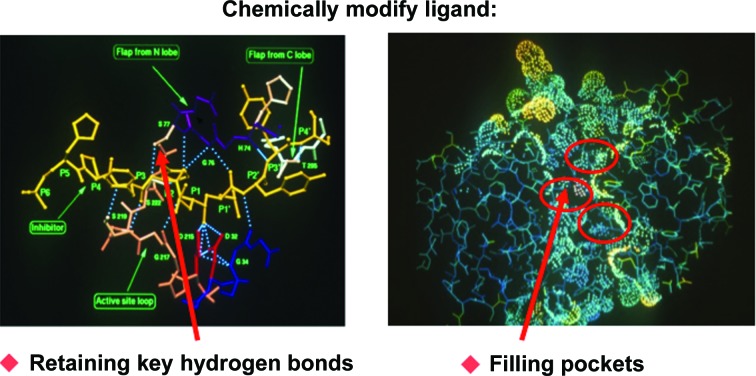
Designing renin inhibitors in the 1980s based on models of renin (Blundell *et al.*, 1983 ▸). This was achieved using interactive graphics, maintaining the hydrogen bonds and filling the specificity pockets.

These studies became increasingly central as knowledge of the pepsin superfamily, the aspartic proteinases, expanded in the 1970s with the identification of renin, which is involved in the first step of the pathway that controls blood pressure, involving the proteolysis of angiotensinogen to give angiotensin 1 (Atkinson, 1980 ▸; Skeggs *et al.*, 1980 ▸). This became a major target for antihypertensive drugs. A three-dimensional model of renin, developed on the basis of the structures of fungal enzymes with the computer program *FRODO* (Jones, 1978 ▸) on an Evans and Sutherland interactive molecular graphics (Blundell *et al.*, 1983 ▸), was used by many pharmaceutical companies in their drug discovery. This was achieved by using emerging knowledge of the aspartic proteinase mechanism and ideas about the transition state derived from the fungal pepsins (Tickle *et al.*, 1984 ▸; Cooper *et al.*, 1987 ▸; Sali *et al.*, 1989 ▸). Fig. 3 ▸ shows a model of the interactions of renin with the substrate, identifying the subsites on either side of the two active-site aspartates, constructed in the 1980s.

Early inhibitor designs focused on modelling a transition-state analogue, maintaining the hydrogen bonds in synthetic analogues of the substrate angiotensinogen, while modifying the peptide bonds and optimizing the interactions of the synthetic side-chain equivalents, guided by the renin model. This was achieved by continuous interactions between the academic laboratory in Birkbeck with the group of Michael Szelke (Cooper *et al.*, 1987 ▸) and the renin medicinal chemistry group at Pfizer Groton (Sali *et al.*, 1989 ▸). Eventually, structures of renins and their complexes with substrate analogues became available (Rahuel *et al.*, 1991 ▸; Dhanaraj *et al.*, 1992 ▸), which allowed more reliable structure-guided discovery. Thus, the process of structure-guided design of renin inhibitors from the late 1970s through to the early 1990s involved an exchange of knowledge with companies including Merck Sharp & Dohme, Pfizer, Glaxo, Wellcome, Zeneca and many others.

### 4.2. HIV protease and AIDS antivirals   

A few years after the first efforts to use structure-guided design for renin inhibitors, further aspartic proteinases became the focus of international attention; these were the retroviral proteases, in particular the human immuno­deficiency virus (HIV) protease that facilitated the impressive development of AIDS antivirals. In 1978 we had proposed with the groups of Jordan Tang and Mike James (Tang *et al.*, 1978 ▸) that the pepsin family of enzymes had evolved from an ancestral dimer to a single polypeptide chain through gene duplication, fusion and divergence. The evidence was the repeated occurrence of the Asp-Thr-Gly motif in the amino-acid sequence of the pepsins and the close resemblance of the three-dimensional folds of the two halves of the pepsin structures that are related by a pseudo-dyad symmetry. Six years later the sequence motif was observed in the retroviral proteases, first in *Rous sarcoma virus* and then in HIV soon after the AIDS epidemic was recognized in the USA and Europe (Toh *et al.*, 1985 ▸); the overall sequence was consistent with its being a relative of the predicted ancestral dimeric aspartic protease.

Several groups embarked on defining the structures of the retroviral proteases. A model was produced by Pearl & Taylor (1987 ▸) based on the aspartic proteinase evolutionary relationship. In 1989 X-ray structures were defined by Alex Wlodawer and coworkers for *Rous sarcoma virus* protease (Jaskólski *et al.*, 1989 ▸, 1990 ▸) and HIV protease (Navia *et al.*, 1989 ▸), and a partial modification of the structure independently by the Wlodawer and Blundell laboratories (Wlodawer *et al.*, 1989 ▸; Lapatto *et al.*, 1989 ▸). Work in the Blundell laboratory was developed collaboratively with Pfizer (Blundell *et al.*, 1990 ▸), with their work on the expression and characterization of HIV protease and ours on the structure; this collaboration was an exercise in knowledge exchange and sharing!

The resemblance of these putative ancestral dimers to renin suggested that inhibitors similar to those of renins and other pepsin-like enzymes might be effective. This gave encouragement to the development by 1997 of four successful AIDS antivirals (saquinavir from Roche Pharmaceuticals, ritonavir from Abbot, indinavir from Merck and nelfinavir from Agouron). It demonstrated the importance of understanding the genome not only in terms of the functions of gene products, but also of their architectures for use in structure-guided drug discovery, as recorded recently in an excellent history of macromolecular crystallography and its fruits (Jaskolski *et al.*, 2014 ▸).

### 4.3. Automation of comparative modelling   

The renin and HIV protease drug-discovery campaigns underlined the fact that although crystallography was advancing quickly, the percentage of gene products encoded by the human genome with experimental structures was less than 10%. Comparative modelling using the structures of homologues was proving useful in generating reasonable models, although much of the experimental protein crystallography community was sceptical. It was time to establish computational approaches that were accessible to all and could be evaluated objectively. In 1987 we set out our agenda in a paper in *Nature* entitled *Knowledge-based prediction of protein structures and the design of novel molecules* (Blundell *et al.*, 1987 ▸). Our first attempt at Birkbeck was to assemble a model from fragments of the structures of homologous proteins; a talented PhD student, Mike Sutcliffe, developed the computer program *COMPOSER* (Sutcliffe, Hannif *et al.*, 1987 ▸; Sutcliffe, Hayes *et al.*, 1987 ▸). The software was commerialized by Tripos; it was widely used in industry, but was also made freely accessible to academic groups.

The second approach was stimulated by software called *RESTRAIN*, used to refine protein structures in X-ray crystallography, developed by a colleague, David Moss, at Birkbeck. In this software, knowledge of protein geometry, including interatomic distances and group planarity, was used to restrain the models refined against the electron density. Our initial thought was to exploit restraints from knowledge of the structures of protein homologues to optimize models, exploiting distance geometry as used in NMR structure analysis. Andrej Sali, then a graduate student in the group, radically reformulated this suggestion in the program *MODELLER* (Sali & Blundell, 1993 ▸), an approach to satisfying restraints from knowledge of homologues that has proved user-friendly and popular, at the time of writing having over 9500 citations in the literature.

The other challenge with respect to comparative/homology modelling was the need to select templates to model the proteins, a process usually termed ‘fold recognition’. David Eisenberg and coworkers produced the first example of ‘threading’ a sequence through the structure of a homologue (Bowie *et al.*, 1991 ▸); in parallel, David Jones, Willie Taylor and Janet Thornton (Jones *et al.*, 1992 ▸) developed a similar, also widely used, computer program called *THREADER*. Our approach depended on amino-acid environment-specific substitution tables to evaluate sequence and structure compatibility (Overington *et al.*, 1990 ▸, 1992 ▸); it first appeared as *QSLAVE* (Johnson *et al.*, 1993 ▸; Blundell & Johnson, 1993 ▸), but was later updated and rewritten as *FUGUE* (Shi *et al.*, 2001 ▸).

## 5. Exploring biological and chemical space   

**Figure 4:**
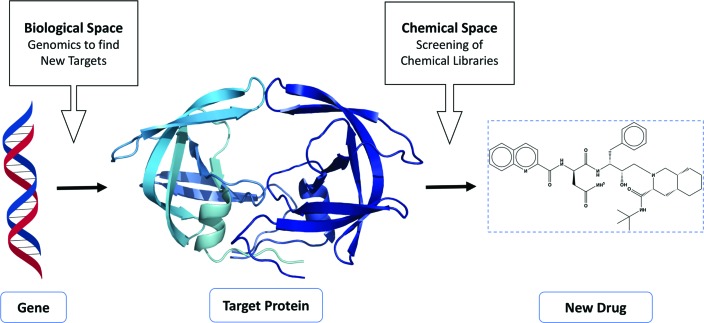
Schematic to illustrate the concept of drug discovery by exploring biological space encompassing genome information to identify new targets, followed by exploration of chemical space using screening libraries based on knowledge of the target protein structure. The target protein in the illustration is HIV-1 proteinase (PDB entry 3phv) and the drug is HIV-1 proteinase inhibitor (PDB entry 9hvp). The figure is reproduced with permission from Thomas *et al.* (2017 ▸).

The development of HIV protease inhibitors for use as AIDS antivirals represented a new paradigm in structure-guided drug discovery. It underlined the importance of understanding the proteome in order to identify targets if the genome sequence is known; this would involve defining the structures of gene products or ‘exploring biological space’. The story of the development of HIV protease inhibitors also emphasized the importance of screening the putative drug target with small molecules to identify chemical entities that might bind; this is ‘exploring chemical space’. This has become a major challenge three decades later when genomics has advanced and we have available not only ‘the human genome sequence’ but also the possibility of sequences of the genomes of many individuals, leading to personalized medicine. We also have thousands of sequences of strains of infectious agents such Mycobacteriaceae that give rise to tuberculosis and leprosy, in principle allowing the selection of appropriate combination therapies. This idea of drug discovery can be summarized as in Fig. 4 ▸.

### 5.1. Biological space and ligandability   

The interest of Peter Goodford in haemoglobin ligands (see above) led him to devise the computer program *GRID* (Goodford, 1985 ▸), which uses the interaction of a probe group together with a protein of known structure; energy values are computed at grid positions throughout and around the macromolecule. Probes include water, the methyl group, amine nitrogen, carboxyl and hydroxyl. The energies are contoured and used to identify ligand-binding clefts for drug design. In 1984 Goodford founded Molecular Discovery Ltd, a software company working in the area of drug discovery with the aim of providing *GRID* software. This enabled one of the first examples of rational drug design with the discovery in 1989 of zanamivir against influenza virus by Peter Colman and Joseph Varghese at the Australian CSIRO.

Binding-site ligandability is usually assessed from the three-dimensional structures of possible target proteins in terms of the concavity of putative binding sites using *PocketFinder*, which is based on *LIGSITE* (Hendlich *et al.*, 1997 ▸), *Pocket­Depth* (Kalidas & Chandra, 2008 ▸) and *fpocket* using Voronoi tessellation (Le Guilloux *et al.*, 2009 ▸). Many other approaches investigate interaction energies, for example by using a van der Waals probe to explore the protein-binding site as in *Q-SiteFinder* (Laurie & Jackson, 2005 ▸), or using random-forest classifiers and residue-based properties as in *SitePredict* (Bordner, 2009 ▸). Schmidtke & Barril (2010 ▸) showed that a combination of such approaches identified 95% of the binding sites of known protein–ligand structures.

### 5.2. Understanding chemical space   

For a time in the late 1980s and early 1990s, it seemed that structure-guided approaches might become redundant. Chemical libraries grew in size as companies expanded their internal compound collections and bought in compounds, often from natural sources. Combinatorial chemistry, the combining of series A and series B compounds using a simple generic chemistry, offered the prospect of millions of new compounds, and the roboticization of screening assays seemed to complete a formidable armory in the large pharmaceutical companies. ‘Chemical space’ would be explored and conquered!

However, the number of new drugs approved by the Food and Drug Administration (FDA) did not increase. Awareness that the number of possible chemical compounds might be as great as 10^80^ began to cause increased concern. Lipinski and coworkers suggested a ‘rule of five’ (Lipinski *et al.*, 2001 ▸) from an analysis of drug candidates that made it to the market, taking into account the drug pharmacokinetics in the human body, including absorption, distribution, metabolism and excretion (ADME):

(i) no more than five hydrogen-bond donors (the total number of nitrogen–hydrogen and oxygen–hydrogen bonds);
(ii) no more than ten hydrogen-bond acceptors (all N or O atoms);
(iii) no more than five rotatable bonds;
(iv) a molecular mass of less than 500 Da;
(v) an octanol–water partition coefficient log*P* not greater than 5.

Even these additional restrictions together with a maximum of four rings leads to an estimate of 10^63^ compounds (Bohacek *et al.*, 1996 ▸). Chemical libraries of several hundred thousand drug-like compounds explored only a tiny area of the chemical space!

## 6. Emergence of fragment-based drug discovery   

One of the most interesting approaches to reducing the size of chemical space was to decrease the complexity of the chemicals screened, for example by using smaller chemical entities with molecular weights of <300 Da: so-called fragments. This increases the promiscuity in binding targets and allows a decrease in the size of the chemical screening library, but still gives rise to well defined and high-quality directional interactions. The affinity of fragments for ‘hotspots’ arises from displacing ‘unhappy’ water molecules (Hajduk, Huth & Fesik, 2005 ▸; Hajduk, Huth & Tse, 2005 ▸; Ichihara *et al.*, 2014 ▸), leading to high ligand efficiency (Murray *et al.*, 2014 ▸; Arnold, 2014 ▸). The approach is known as fragment-based drug discovery.

Early experiments at Abbott used ligand-based NMR (Shuker *et al.*, 1996 ▸; Hajduk & Greer, 2007 ▸; Harner *et al.*, 2013 ▸) to detect binding. However, selectivity is gained most efficiently by using structure-guided methods to first define the position of the bound fragment, before growing it or linking it to other fragments. This was achieved by X-ray crystal screening (Blundell *et al.*, 2002 ▸; Murray *et al.*, 2014 ▸; Murray & Blundell, 2010 ▸), developed at Astex, a company founded by Harren Jhoti, Chris Abell and Tom Blundell in 1999. This company was funded by Abingworth Investments, and was established for the first year with three postdocs in the Blundell and Abell laboratories at Cambridge University, where it was shown that binding could be defined structurally using X-ray analysis by soaking fragments into crystals. After this had been demonstrated, Harren Jhoti, who had previously been at GSK and had established himself at the Grafton Centre in the central shopping area of Cambridge, led the team as Chief Scientific Officer to the Science Park, where Tim Haines, an experienced entrepreneur, joined as CEO. Astex initially screened several hundred fragments using high-throughput X-ray analysis of cocktails of six to ten fragments soaked into apoprotein crystals. Cocktails providing hits were then examined by soaking each fragment separately to confirm the binder, which could often be recognized from the initial crystal screen electron density, if the fragments in each cocktail were sufficiently structurally diverse.

Fragment-based screening is exploited with small chemical libraries (Congreve *et al.*, 2008 ▸; Hajduk & Greer, 2007 ▸), perhaps with as few as 1000 compounds of molecular weight less than 300 Da that are consistent with the ‘rule of three’ developed by Astex (Congreve *et al.*, 2003 ▸). Ligand efficiency of fragments is often high, as the atoms can be involved in productive interactions as stated by Hann’s complexity rule (Hann *et al.*, 2001 ▸). A high-affinity lead molecule thus developed from a fragment hit retains the key binding interactions of the original fragment with the ‘hotspot’ on the target protein.

Ichihara *et al.* (2014 ▸) showed that fragment binding exploits a distinct difference between the thermodynamic profiles of the water molecules displaced by fragment hits and those displaced by the optimized lead compounds derived from them. Fragments tend to displace water molecules with un­favourable entropies; these are constrained in their configurations relative to those displaced when fragments are grown during lead optimization. In this author’s experience this often corresponds to a region with small lipophilic patches adjacent to polar groups, thereby providing a restriction on the orientations that the water molecule may adopt.

Chris Radoux at the Cambridge Crystallographic Data Centre, the Blundell laboratory and UCB-Celltech, a pharma company, have developed a hotspot-mapping program (Radoux *et al.*, 2016 ▸). This carries out a global grid-based search of the protein structure, using three five-atom ring fragments, one with a hydrogen-bond donor, another a hydrogen-bond acceptor and a third a lipophilic methyl group, to create corresponding donor, acceptor and hydrophobic hotspot maps. These are then weighted by a depth factor that tends to give a good estimate of the likelihood of there being an unhappy water.

Advances in protein crystallography using high-throughput, roboticized approaches at synchrotrons offer further opportunities. Frank von Delft and coworkers have developed automated methods to soak crystals with fragments and mount them in the X-ray beam. Perhaps the most impressive advance has been *PanDDA* (Pearce *et al.*, 2017 ▸), a method that reveals electron density for only the changed state, even from poor models and inaccurate maps, by subtracting a proportion of the apo state, accurately estimated by averaging many apoprotein crystals.

**Figure 5:**
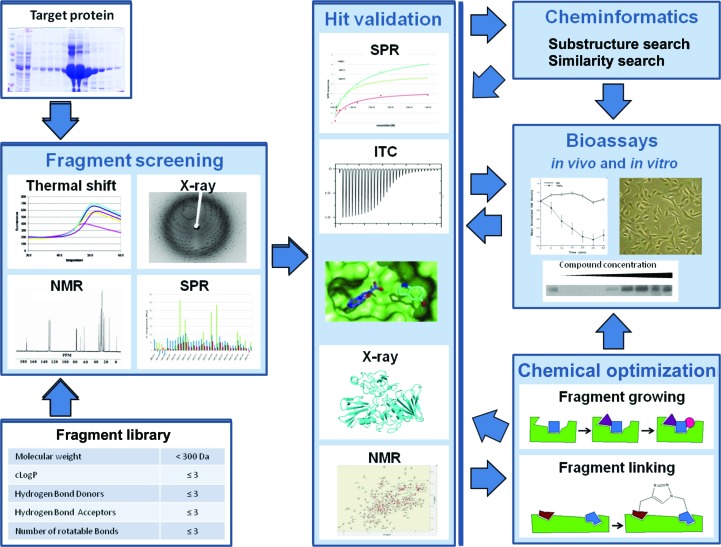
Fragment-based drug discovery: fragment screening, validation and chemical optimization through iterative growth and/or linking.

The relatively low affinities with which fragments bind to proteins mean that a combination of biochemical, biophysical and structural techniques must be used to monitor hit identification, validation and subsequent elaboration into lead molecules. Many groups use a two-stage approach of high-throughput screening of the fragment library using fluorescence-based thermal shift measurements (Fig. 5 ▸; Scott *et al.*, 2012 ▸; Niesen *et al.*, 2007 ▸), ligand-based NMR, surface plasmon resonance (SPR) and, increasingly with the roboticized screening facilities available on synchrotron beamlines, X-ray crystallographic screening. The fragment hits that are common between these techniques are then validated by optimization of the resolution of the structures by X-ray diffraction or structure determination by NMR, as well as definition of the kinetics using SPR and of the thermodynamics of the binding using isothermal calorimetry.

Knowledge of the structure of the complex of the fragment with a target protein allows the initial use of nonchiral fragments, which are optimized using structure-guided approaches to make specific interactions and to introduce chirality into the molecules. The validated fragment hits are then elaborated iteratively by growing to a larger molecular weight or by linking using structure-guided techniques.

## 7. Fragment-based drug discovery in oncology and infectious disease   

### 7.1. Cancer drugs reach the market   

Fragment-based drug discovery developed during the mid-to-late 1990s in both large pharma such as Abbott (Shuker *et al.*, 1996 ▸) and small biotechs, for example Sunesis and Astex. Scientists trained in universities, institutes, small biotechs and large pharma, together with serial entrepreneurs, all played roles in these startups; those from pharma often expressed their expectation of having a little more influence on new developments than in the large international companies where they were trained. In small companies such as Astex, those from large pharma often chose classical targets such as protein kinases to test the new technolologies. Thus, Astex has worked on a range of protein kinases where the targets had already been validated by big pharma. Other large companies established structure-guided fragment-based drug discovery in-house, but in parallel outsourced work to small companies. Astex had significant investments of the order of $30 million from each of Novartis, Jannsen, AstraZeneca and GSK. This also encouraged co-development to move drug candidates through Phases 2 and 3 to gain FDA approval.

These features of development have characterized the first three drugs to reach the market that have exploited fragment-based drug discovery. Thus, in 2011 the first fragment-derived drug, vemurafenib, was approved, targeting a mutant form of BRAF and extending life for patients with skin cancer. This was discovered at Plexxikon, a small company founded in 2003, and developed in partnership with Roche. The second, venetoclax, developed by AbbVie and Genentech, binds to BCL-2 and blocks its interaction with other proteins; this gained FDA approval in 2016 for chronic lymphocytic leukaemia (CLL). In 2017, ribociclib, developed by Astex and Novartis to target the protein kinase Cdk4, was approved for use as a first-line treatment for advanced breast cancer, in combination with letrozole.

### 7.2. Targeting protein–protein interactions   

In the early 2000s, structure-guided fragment-based drug discovery was often spun back from biotechs and large pharma into universities and institutes. In the case of Astex this was motivated by the fact that pharma tended to select targets from large enzyme superfamilies such as protein kinases (Zhang *et al.*, 2009 ▸) with well defined concave active sites that have been found to be ‘druggable’. However, it had become increasingly evident that it is difficult to obtain selectivity, especially with transition-state and intermediate-state analogues of enzymes or those targeting cofactor-binding sites.

The challenges of selectively targeting a particular protein kinase became very clear as the numbers of superfamily members that were easily assayed increased from less than 20 in the 1990s to hundreds a decade later. Much of the optimism in obtaining very good selectivity with protein kinase inhibitors by exploiting subpockets around the ATP-binding site has been moderated by the discovery that subclusters of protein kinases with similar cofactor-binding sites are recognized by many of the molecules previously thought to be selective.

One of the ways of improving selectivity is to move away from targeting active sites towards regulatory multiprotein systems that are critical to cell activity (Blundell & Srinivasan, 1996 ▸). Cell-surface receptors such as the FGF or Met receptors, intracellular signalling pathways involving protein kinases, and nuclear regulatory systems, for example mediating DNA double-strand break repair, are all regulated over space and time by multi-component assemblies that not only co-locate various critical components but are also likely to play a role in increasing signal to noise through cooperative assembly. Simple binary interactions between two proteins would often occur opportunistically in the cell, especially in the cell membrane or in the limited environment of the nucleus or cytoplasm. However, a weak binary interaction followed by interactions with further components would give a cooperative but reversible assembly of a large multicomponent complex, allowing selective signalling regulation in the cell (Bolanos-Garcia *et al.*, 2012 ▸). There are some occasions where binary systems are essential in signalling and regulatory processes, and these are often mediated by the concerted folding and binding of one protein onto the other. This was recognized more than 40 years ago in polypeptide hormones such as glucagon, which are disordered in solution but can associate with the receptor by folding and binding in a concerted manner (Sasaki *et al.*, 1975 ▸). They often first bind through an anchor residue, which binds in a pocket of a globular protein, the hotspot of the interaction.

Such concerted folding and binding is found widely in intracellular systems, for example the breast cancer susceptibility protein BRCA2, which controls the function of RAD51, a recombinase enzyme, in pathways for DNA repair by homologous recombination. The interaction of the BRCA2–BRC4 motif with RAD51 involves a phenylalanine, the anchor residue, which recognizes a well defined pocket on RAD51 (Pellegrini *et al.*, 2002 ▸). This then also allows a much smaller pocket, a second hotspot, to be recognized by alanine in the sequence F*XX*A, the interaction probably being driven by unhappy waters, leading to high selectivity. The remaining part of the BRC4 repeat then folds onto the surface of RAD51 as a helix onto a shallow groove. The cooperative folding and binding provides a second mechanism for obtaining selectivity and has been widely studied for intracellular systems by Dyson & Wright (2002 ▸). Exploitation of these anchor sites or hotspots has been used in the design of inhibitors (Meireles *et al.*, 2010 ▸; Koes & Camacho, 2012 ▸). Systematic analysis in our laboratory of over 9000 pairwise non-overlapping protein–protein interfaces, organized in our databases and filtered for structure quality, has indicated that protein–peptide interfaces make more extensive use of concavity than other kinds of interfaces, both on average and at their deepest (Jubb *et al.*, 2015 ▸).

**Figure 6:**
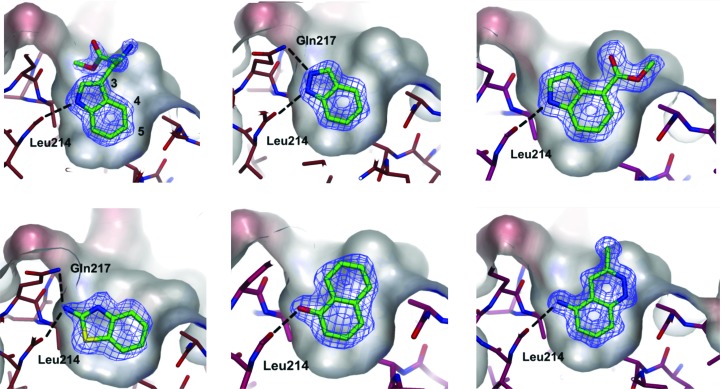
Targeting the interaction between the tumour suppressor BRCA2 and the recombination enzyme RAD51. Protein engineering was used to create a monomeric form of RAD51 by humanizing a thermostable archaeal orthologue, RadA, known as MAYM RadA, and using this protein for fragment screening. View through the Phe pocket of crystal structures of validated fragments. Weighted 2*mF*_o_ − *DF*_c_ electron-density maps of the partially refined structures are calculated before inclusion of ligands. This figure was created by Dr Marko Hyvönen using material published in Scott *et al.* (2013 ▸).

In 2006 the Wellcome Trust established a new programme of Translation Awards to encourage the translation of their funded basic science. One of the awards was a programme on targeting the interaction of the BRCA2–BRC4 motif with RAD51 (Pellegrini *et al.*, 2002 ▸). This proved to be an excellent proof of principle for biophysical fragment-based drug-discovery approaches to target a protein–protein interface, especially as the shallow binding site with relatively small pockets was predicted to be undruggable by any of the retrospective approaches to assess this. Disruption of this interaction *in vivo* is hypothesized to give rise to cellular hypersensitivity to radiation and genotoxic drugs. We used protein engineering to create a monomeric form related to RAD51, known as MAYM RadA, by humanizing a thermostable archaeal orthologue, RadA, for use in fragment screening (Moschetti *et al.*, 2016 ▸). Initial screening of a fragment library by thermal shift, followed by validation using NMR and X-ray crystallo­graphy, resulted in the structures of approximately 80 fragments bound to the humanized surrogate of RAD511, which disrupted the interaction with BRCA2 (Scott *et al.*, 2013 ▸); see Fig. 6 ▸ for examples.

The growth of the fragments bound to RAD51 was guided by the co-crystallized structures together with the structure of RAD51 in complex with the BRC4 region of BRAC2, and was able to improve the *K*_d_ from the millimolar to the sub­millimolar range (Scott *et al.*, 2013 ▸). We developed indole-based fragments that bind in the shallow surface pocket of the humanized surrogate of RAD51, developing small-molecule inhibitors that are approximately 500-fold more potent than the initial fragments. The lead compounds were shown to compete with the BRCA2-derived Ac-FHTA-NH2 peptide and the self-association peptide of RAD51, but they had no effect on ATP binding (Scott *et al.*, 2015 ▸).

### 7.3. Targeting infectious disease   

In 2007, the Astex team were invited to join the Bill and Melinda Gates Foundation initiative called Integrated Methods for Tuberculosis (IMTB) Drug Discovery to develop the use of fragment-based drug discovery for the treatment of tuberculosis. However, after discussion, Astex decided that the focus on oncology in the company should be maintained, and suggested that Chris Abell and Tom Blundell would use the company’s fragment-based drug-discovery approach in the university. The two primary objectives were to ‘substantially improve our ability to discover, identify and validate targets linked with TB persistence’ and ‘to expand our capacity to find and optimize small-molecule inhibitors of validated targets’. This involved evaluating novel technologies that have not previously been focused on tuberculosis drug discovery in a concerted way.

Although target-based campaigns have identified a number of leads that show high potency *in vitro* for targeting *Mycobacterium tuberculosis*, most did not show any translation to an *in vivo* effect. This is likely to be owing to the selection of molecules that are compliant with the rule of five, with smaller and larger molecules often omitted. Multiple replication states of *M. tuberculosis*, together with many lesions that differ in local environments, are likely to be present in tuberculosis patients, leading to problems of drug penetration. The ability of fragment-based discovery to explore more chemical space makes it an appropriate approach which offers new routes to finding drugs for tuberculosis (Mendes & Blundell, 2017 ▸).

The Abell and Blundell laboratories decided to use structure-guided fragment-based drug discovery to target the pantothenate pathway, for which they already had biochemical, structural and inhibitor studies; this included panto­thenate synthase, for which they had defined the the structure of the *Escherichia coli* enzyme (von Delft *et al.*, 2001 ▸) and developed work targeting the enzyme from *M. tuberculosis* (Silvestre *et al.*, 2013 ▸). They also targeted proteins suggested by other collaborators in the Gates Foundation IMTB or subsequently the Gates HIT-TB and EU-FP7 MM4TB consortia. An example of this is the work on EthR, a member of the TetR family of transcription factors (Frénois *et al.*, 2006 ▸).

**Figure 7:**
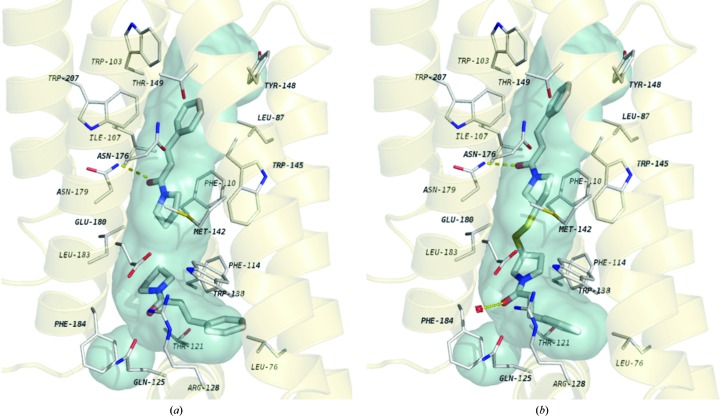
Fragment binding (*a*) and retention of the initial conformation and interactions during fragment linking (*b*) to EthR. Ligands are in stick representation and the EthR ligand-binding channel surface is shown in blue. All atoms follow the CPK colouring scheme. Hydrogen bonds are represented by dashed lines. Reproduced with permission from Surade *et al.* (2014 ▸).

The objective of targeting EthR was to lessen the toxicity of the existing drugs. Ethionamide, a specific inhibitor of mycobacterial cell-wall synthesis, is restricted to use as a second-line treatment owing to its toxicity. Ethionamide is a prodrug, activated by a mycobacterial-specific mono-oxygenase, EthA, which is regulated by EthR. It had been proposed that de-repression of the EthA gene and the consequent increased expression of EthA would result in increased levels of bio-activated ethionamide and a decrease in the minimal inhibitory concentration (MIC) of ethionamide, and this suggested that a way of reducing the effective dose of ethionamide is to target the allosteric site, which binds a highly lipophilic hexadecyl octanoate. This had already been studied structurally with a view to producing inhibitors (Frénois *et al.*, 2006 ▸). Fragment screening identified hydrophilic ligands which formed hydrogen bonds (see Fig. 7 ▸) that were not exploited in the function of this repressor at its hydrophobic allosteric binding site, and these were developed into larger molecules with good affinities *in vitro* (Willand *et al.*, 2009 ▸; Flipo *et al.*, 2012 ▸; Surade *et al.*, 2014 ▸; Nikiforov *et al.*, 2016 ▸). However, these proved to be less useful than the fragments *in vivo*, and the development of fragment-sized EthR ligands with nanomolar minimum effective concentration (MEC) values for the boosting of ethionamide activity in *M. tuberculosis* whole-cell assays has proved a very exciting development (Nikiforov *et al.*, 2017 ▸).

## 8. Structure-guided drug discovery: an example of knowledge exchange in research ecosystems?   

We have seen that during the development of structure-guided drug discovery ideas have originated from all parts of the research ecosystem. From the very early work of Dorothy Crowfoot in the 1930s crystallographers obtained proteins, in her case insulin, from pharmaceutical companies, and in the 1950s she learnt about the optimization of insulin crystallization from the Danish company Novo. The determination of the structure by X-ray crystallography in academia allowed companies to think about new ways of controlling the release of insulin in circulation by using synthetic insulins. Structures of aspartic proteases developed in academia were made possible by the purification of fungal enzymes studied for cheesemaking in industry. The structure of renin was developed and published between the Blundell group and Pfizer with the objective of designing inhibitors that might be antihypertensives. The idea of a dimeric aspartic protease came from an international academic team and was discovered in HIV; it informed the modelling of HIV protease and the design of new HIV antivirals in industry, before structures of HIV protease were produced in both academia and industry. Structure-guided fragment-based discovery was developed in large pharma and biotechs, but has been exploited in academia for the development of new inhibitors targeting protein–protein interactions and also antimicrobials to combat mycobacterial infections such as tuberculosis.

These observations provide a strong argument against the so-called ‘linear model’, where ideas flow only in one direction from academic institutions to industry. They underline the importance of knowledge exchange often in localized areas such as Cambridge, where university spinoffs have included companies based on electronics and IT, followed later by biomedicine. The ‘demonstration effect’ of successful enterprises provides a major stimulus to further company formation by employees of first-generation firms, and the university’s liberal policies facilitated collaborative academic enterprise (Jennings, 1991 ▸; Garnsey, 1995 ▸). Even from the Biochemistry Department Sanger Building and the Gurdon Institute, which share adjacent buildings, colleagues have formed many companies, including Kudos (Steve Jackson), Astex Pharmaceuticals (Blundell and Abell), Biotica (Leadlay), Abcam and Chroma Therapeutics (Tony Kouzarides).

The principal attractors of new entry differ according to the technological area. As shown by Faulkner & Senkar (1995 ▸), biotechnology has the highest level of formal linkage activity to academic centres. It is the only field in which public-sector research contributes more knowledge to R&D than do other companies. This conclusion has been supported by the work of the London Business School group on principal attractors of new entry. In their study of the US computer industry, Swann *et al.* (1998 ▸) demonstrated that new chip companies cluster closely with those concerned with hardware, software and systems research. On the other hand, the principal new entry attractor in US biotechnology tends to be the science base. New high-tech companies in therapeutics, diagnostics and equipment tend to cluster close to centres of excellence in medical and biological research.

What then is the nature of networking and interactions between the science base and high-tech new companies? Faulkner and Senkar describe the linkages as often informal and face to face; good personal relationships are key to successful collaborations and personal interactions are crucial to building mutual trust and respect. These certainly coincide exactly with our own experiences in Cambridge. All of these features need to be kept in mind when government sets up structures to support technological interactions. As Faulkner & Senkar (1995 ▸) comment, overly zealous government programmes often create ‘false marriages’ and undermine informal networking.

Government schemes to network basic scientists with industry need to have their main objective as networking. They must be flexible and able to accommodate changes of emphasis and direction. They need to encourage trust and close personal links so that new ideas can be accommodated. My experience in developing AIDS antivirals is a good example. This started as a collaboration on finding inhibitors that would be useful antihypertensives, but changed direction smoothly and profitably.

In the linear model benefits are assumed to flow in the form of new useful knowledge to be directly incorporated into new products or processes. Martin *et al.* (1996 ▸) contrast this with their model of knowledge exchange, where the contributions from the publicly funded basic research and industrial activity come in the form of small and often largely invisible flows. The development of protein crystallography and structure-guided drug discovery are powerful examples of the importance of knowledge exchange.

Government expectations about the benefits from basic research have changed. A new ‘social contract’ is emerging; there are more specific expectations that basic research should generate economic and social benefits in return for the substantial public funds that it receives. If we can structure research in universities, institutes and government establishments in a way that encourages networks with industry and allows individuals to move freely between academia and industry, then we will have a much better chance of exploiting new technologies. This could generate considerable wealth and improve the quality of our environment and health, not only in the United Kingdom, but also in many other developed and developing countries in the coming years.

In summary, structure-guided drug discovery is a story of applications of protein crystallography and knowledge exhange between academia and industry. It has led to new drug approvals by the Food and Drug Administration in the USA and hope for treatment of rare genetic diseases and infectious diseases in the developing world.
